# *Neisseria* species on the tonsillar surface predicts favorable clinical outcomes in patients with Immunoglobulin A nephropathy who underwent tonsillectomy

**DOI:** 10.1080/0886022X.2025.2550617

**Published:** 2025-09-03

**Authors:** Ryosuke Sato, Takahiro Inoue, Risa Wakisaka, Michihisa Kono, Hidekiyo Yamaki, Kenzo Ohara, Takumi Kumai, Kan Kishibe, Naoki Nakagawa, Miki Takahara

**Affiliations:** ^a^ Department of Otolaryngology-Head & Neck Surgery, Asahikawa Medical University, Asahikawa, Japan; ^b^ Department of Innovative Head & Neck Cancer Research and Treatment, Asahikawa Medical University, Asahikawa, Japan; ^c^ Division of Cardiology & Nephrology, Department of Internal Medicine, Asahikawa Medical University, Asahikawa, Japan

**Keywords:** IgA nephropathy, bacterial flora, *Neisseria* sp, tonsillar surface, tonsillectomy

## Abstract

An aberrant mucosal immune response against commensal bacteria in the tonsils is hypothesized to be one of the pathogenic mechanisms underlying immunoglobulin A nephropathy (IgAN). However, the bacteria involved in the pathogenesis of IgAN have not been fully elucidated. In this study, we compared the differences in tonsillar bacterial flora between IgAN (*n* = 101) and recurrent tonsillitis (RT) (*n* = 117) based on swab cultures from tonsillar surfaces and the center of the tonsils. The association between *Neisseria* sp. presence in the tonsils and the clinical course of patients with IgAN who underwent tonsillectomy (*n* = 81) was investigated. Significant differences in the detection rates of *Neisseria* sp. on tonsillar surfaces were observed between the IgAN and RT groups (88.0 and 74.4%, respectively). Among the 81 patients, the rates of clinical remission, disappearance of proteinuria, renal function preservation, and renal survival were significantly higher in patients with *Neisseria* sp. present on the tonsillar surface than in those without. The Cox proportional hazards model indicated the presence of *Neisseria* sp. on the tonsillar surface as an independent favorable factor for the rates of clinical remission, decreasing renal function, and renal death (hazard ratios: 3.42, 0.03, and 0.06, respectively). However, there were no significant differences in the clinical course, except for renal survival rates, between patients with and without *Neisseria* sp. at the tonsillar center. This study revealed *Neisseria* sp. was more frequently found on the tonsillar surface of patients with IgAN than those with RT, and their presence was linked to better clinical outcomes following tonsillectomy.

## Introduction

Immunoglobulin A nephropathy (IgAN) is the most prevalent form of primary glomerulonephritis. The long-term renal prognosis is unfavorable, with ∼40% of patients with IgAN progressing to renal failure within 20 years of diagnosis [1]. Immunoglobulin A (IgA) is the predominant antibody isotype in the mucosal immune system for extrinsic antigens [2]. However, in IgAN, overproduction and abnormal formation of IgA cause renal damage through the deposition of IgA complexes in the kidneys [3]. As serum IgA levels increase and urinary findings worsen due to acute tonsillitis, tonsillectomy is considered a potential treatment for IgAN [4,5]. Several studies have reported that tonsillectomy increases the clinical remission and renal survival rates in IgAN [6–8]. These findings suggest that the tonsils are a source of IgA that contribute to the pathogenesis of IgAN.

We previously reported that deoxycytidyl-deoxyguanosine oligodeoxynucleotide (CpG-ODN), a conserved bacterial DNA, enhanced IgA production by tonsillar mononuclear cells obtained from patients with IgAN through B-cell-activation factor and a proliferation-inducing ligand [9,10]. Another study reported that the mucosal administration of CpG-ODNs in mice induced the production of IgA in the sera and upper respiratory tracts [11]. In this context, an aberrant mucosal immune response against commensal bacteria in the tonsils is hypothesized to underlie IgAN pathogenesis. However, the key bacteria implicated have not yet been fully elucidated. This study compared the differences in tonsillar bacterial flora between IgAN and recurrent tonsillitis (RT) using swab cultures from the tonsillar surface and center. Additionally, we examined whether the presence of *Neisseria* sp. affected the clinical course of patients with IgAN after tonsillectomy. The study revealed that the detection rate of *Neisseria* sp. on the tonsillar surface was significantly higher in patients with IgAN than in those with RT; furthermore, patients with *Neisseria* sp. exhibited a favorable clinical course after tonsillectomy.

## Methods

### Methods of data collection

We retrospectively examined the tonsillar flora of patients with IgAN diagnosed through renal biopsy who underwent tonsillectomy at Asahikawa Medical University Hospital between January 2001 and December 2022. At our institution, all patients were informed about the tonsillectomy procedure, and steroid pulse therapy was administered when deemed necessary by the physician according to previously reported protocols [12]. The positivity rates of bacteria on the tonsillar surface and at the tonsillar center in patients with IgAN were compared to those in control patients with RT. We further investigated whether the presence of a specific bacterium in the tonsils influenced the clinical course of patients with IgAN after tonsillectomy. The exclusion criteria were estimated glomerular filtration rate (eGFR) < 30 (mL/min/1.73 m^2^) or insufficient clinical information in the patient’s medical records.

### The recruitment of patients

During the study period, 101 patients with IgAN underwent tonsillectomy, and their tonsillar bacterial flora was compared with that of 117 patients with RT. Subsequently, 17 patients with IgAN were excluded due to incomplete clinical information in their medical records, and 3 patients were excluded due to eGFR < 30 mL/min/1.73 m^2^. As a result, a total of 81 patients were included in the analysis of clinical courses (Supplemental Figure S1).

### Methods of evaluating tonsillar flora

The tonsillar flora at the two distinct tonsil sites was assessed following sterile cotton swab culture. Before surgery, while the patients were under general anesthesia, the tonsillar surface was wiped with a sterile cotton swab. After tonsillectomy, the resected tonsil was immediately bisected, and the center of the tonsil was swabbed. All swab samples were transported to the Asahikawa Medical University Hospital Microbiology Laboratory using BD BBL™ CultureSwab™ Plus (BD Diagnostics, USA). Swabs were simultaneously plated onto blood agar and chocolate agar. Blood agar plates were incubated aerobically at 35 °C for 48 h. Chocolate agar plates were incubated in a 5% CO_2_ atmosphere at 35 °C for 48 h. Bacterial species were identified using conventional phenotypic methods, including colony morphology, hemolysis patterns, and Gram staining characteristics.

### The associations between the presence of bacteria and clinical course of IgAN

Results of proteinuria and hematuria by urinary qualitative/semi-quantitative examination were converted into scores as follows: (−) to 0, (±) to 0.5, (1+) to 1, (2+) to 2, (3+) to 3, and (4+) to 4. Scores of 1–4 were considered positive for proteinuria or hematuria, whereas scores of 0–0.5 were considered negative. Clinical remission was defined as negative proteinuria and hematuria on two consecutive examinations. Decreased renal function and renal death were defined as a 30% decline in the eGFR and the need for renal replacement therapy, respectively. The duration of clinical remission, decreased renal function, and renal death was defined as the period from tonsillectomy to the occurrence of the respective event. The eGFR was calculated using the equation recommended by the Japanese Society of Nephrology [13]. Because pathological information was not available for all patients, the Oxford classification was assessed in 23 patients, while the histological grade, as defined by the Japanese clinical guidelines for IgAN [14], was assessed in 60 patients.

### Immunohistochemical staining

Immunohistochemical staining was used to analyze the presence of *Neisseria* sp. in renal biopsy specimens from patients with or without *Neisseria* sp. on the tonsillar surface. The mouse monoclonal Ab against *Neisseria gonorrhoeae* (1:400 dilution, sc-57931; Santa Cruz Biotechnology, USA) and the rabbit polyclonal Ab against *Neisseria meningitidis* (1:1000 dilution, GTX36557; GeneTex, USA) served as primary antibodies. The Ventana Benchmark GX (Roche Diagnostics, Switzerland) was used for immunostaining.

### Statistical analysis

The detection rate of each bacterium in the IgAN and RT groups at each site was compared using Fisher’s exact test. Patient characteristics in the presence and absence of a specific bacterium were compared using Fisher’s exact test for categorical variables and the unpaired *t*-test for continuous variables. The rates of clinical remission, negative conversion rates for hematuria or proteinemia, renal function preservation rates, and renal survival rates were compared using Kaplan–Meier analysis and the log-rank test. The Cox proportional hazards models were employed to investigate the factors related to clinical remission, renal function preservation rates, and renal survival rates. The following variables were included as explanatory variables: age ≥ 40 years, use of renin-angiotensin system inhibitors, eGFR ≥ 60 mL/min/1.73 m^2^, urinary protein excretion ≥ 0.5 g/day, administration of steroid pulse therapy, and the presence of *Neisseria* sp. on the tonsillar surface. Due to the limited number of events, we used a stepwise selection method based on the area under the curve for the analyses of renal function preservation and renal survival rates, using the same set of explanatory variables. EZR (Saitama Medical Center, Japan) was used for a stepwise selection method. GraphPad Prism 9 (GraphPad Software, San Diego, CA, USA) was used for all other analyses. Statistical significance was set at *p* < 0.05.

### Ethics

This study was conducted in accordance with the principles embodied in the Declaration of Helsinki. The study design was approved by the Ethics Review Board of Asahikawa Medical University (approval no. 224-8). Written informed consent was obtained from all participants.

## Results

**Figure 1.**
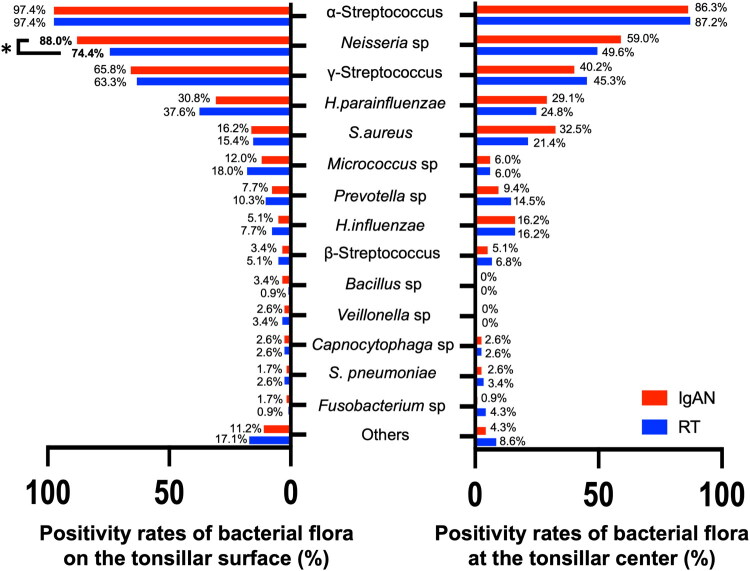
Positivity rates of each bacterium on the tonsillar surface and at the tonsillar center in Immunoglobulin A nephropathy (*n* = 101) and recurrent tonsillitis (*n* = 117). A total of 21 bacteria were detected using the swab culture method. α-*Streptococcus*, *Neisseria* sp., γ-*Streptococcus*, *H. parainfluenzae*, and *S. aureus* were commonly detected both on the tonsillar surface and at the tonsillar center. Fisher’s exact test revealed that the positivity rate of *Neisseria* sp. on the tonsillar surface was significantly higher in the IgAN group than in the RT group. The positivity rates of *Neisseria* sp. in IgAN and RT on the tonsillar surface were 88.0 and 74.4%, respectively, whereas those at the tonsillar center were 59.0 and 49.6%, respectively. *Statistically significant. *H. influenzae: Haemophilus influenzae*; *H. parainfluenzae: Haemophilus parainfluenzae*; IgAN: immunoglobulin a nephropathy; RT: recurrent tonsillitis; *S. aureus: Staphylococcus aureus*; *S. pneumoniae: Streptococcus pneumoniae.*

A total of 218 patients, comprising 101 with IgAN and 117 with RT, were included in the analysis of bacterial flora on the tonsillar surface and at the tonsillar center. The detection rates of bacteria on the tonsillar surface and at the tonsillar center are illustrated in Figure 1. A total of 21 bacteria were detected in the swab culture tests. α-*Streptococcus* was most frequently detected in both IgAN and RT groups. The detection rates of α-*Streptococcus* on the surface were 97.4% for both the IgAN and RT groups, whereas at the center, the detection rates were 86.3 and 87.2%, respectively. The second most frequently detected bacterium was *Neisseria* sp. The detection rates of *Neisseria* sp. on the tonsillar surface in the IgAN and RT groups were 88.0 and 74.4%, respectively, while at the center, they were 59.0 and 49.6%, respectively. Subsequently, γ-*Streptococcus*, *Haemophilus parainfluenzae*, and *Staphylococcus aureus* were commonly detected at both sites. Fisher’s exact test revealed that the detection rate of *Neisseria* sp. on the surface was significantly higher in the IgAN group than in the RT group. No significant differences in the detection rates of other bacteria at each site were observed between the IgAN and RT groups.

**Figure 2.**
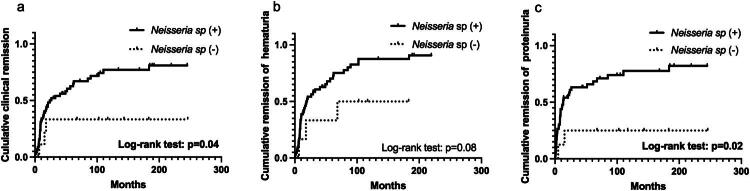
Comparison of remission rates of urinary findings in patients with immunoglobulin a nephropathy positive and negative for *Neisseria* sp. on the tonsillar surface. Kaplan–Meier curves for rates of clinical remission (a) (*n* = 81), hematuria remission (b) (*n* = 71), and proteinuria (c) (*n* = 62). Log-rank tests revealed that the clinical remission rates and remission rates of proteinuria were significantly higher in patients who were positive for *Neisseria* sp. on the tonsillar surface than in those who were negative.

**Figure 3.**
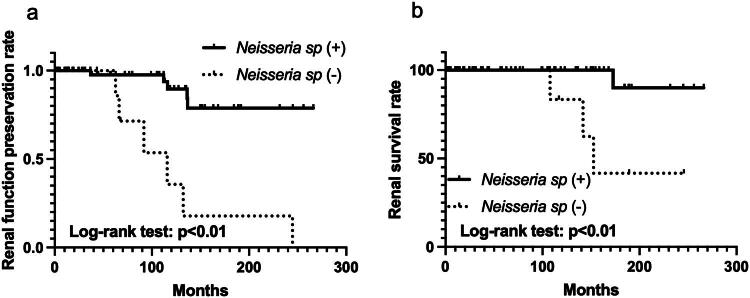
Comparison of long-term outcomes in patients with immunoglobulin a nephropathy positive and negative for *Neisseria* sp. on the tonsillar surface who underwent tonsillectomy (*n* = 81). Kaplan–Meier curves for renal function preservation rates (a) and survival rates (b). Log-rank tests revealed that renal function preservation and survival rates were significantly higher in patients positive for *Neisseria* sp. on the tonsillar surface than in those who were negative.

**Figure 4.**
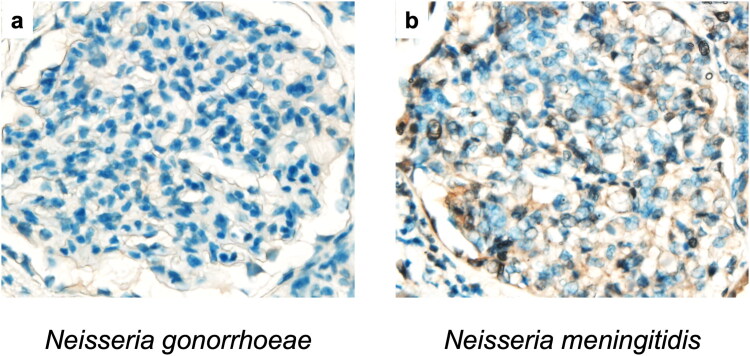
Representative immunohistochemical images of (a) *Neisseria gonorrhoeae* and (b) *Neisseria meningitidis* in renal tissue specimens. All seven patients showed negative staining for *N. gonorrhoeae* and weak to moderate staining for *N. meningitidis* in the glomeruli, regardless of the presence of *Neisseria* sp. on the tonsillar surface.

We subsequently investigated the relationship between the presence of *Neisseria* sp. on the tonsillar surface and the clinical course of patients with IgAN after tonsillectomy. After selection based on the exclusion criteria, 81 patients were enrolled in this study. *Neisseria* sp. was present on the tonsillar surface of 71 patients (87.7%) but was absent in 10 patients (12.3%). Differences in characteristics between patients in the *Neisseria*-positive (*n* = 71) and *Neisseria*-negative (*n* = 10) groups are shown in Table 1. No significant differences were observed between the positive and negative groups. Short- and long-term outcomes of tonsillectomy in patients with IgAN are shown as Kaplan–Meier curves in Figures 2 and 3, respectively. The 3-year clinical remission rates in the positive and negative groups were 54.1 and 33.3%, respectively (Figure 2(a)). The negative conversion rates of hematuria and proteinuria were 60.6 and 33.3% (Figure 2(b)) and 63.2 and 25.0% (Figure 2(c)), respectively. Log-rank tests revealed that clinical remission and negative conversion rates of proteinuria were significantly higher in the positive group than in the negative group. According to the Cox proportional-hazards model, the administration of steroid pulse therapy and the presence of *Neisseria* sp. on the tonsillar surface were independent factors favoring clinical remission (hazard ratios 2.07 and 3.42, respectively) (Table 2). The 10-year renal function preservation and renal survival rates in the positive and negative groups were 89.6 and 35.7% (Figure 3(a)) and 100 and 83.3% (Figure 3(b)), respectively. Log-rank tests revealed that the renal function preservation and renal survival rates were significantly higher in the positive group than in the negative group. According to the Cox proportional-hazards model, administration of steroid pulse therapy and the presence of *Neisseria* sp. on the tonsillar surface were independent factors associated with decreased renal function (hazard ratios 6.42 and 0.03, respectively) (Table 3). For renal death, only the presence of *Neisseria* sp. on the tonsillar surface was a significant favorable independent factor (hazard ratio of 0.06) (Table 4). Renal tissue specimens were available from 5 patients positive and 2 patients negative for *Neisseria* sp. on the tonsillar surface. All 7 patients showed negative staining for *N. gonorrhoeae* and weak to moderate staining for *N. meningitidis* in the glomeruli, regardless of the presence of *Neisseria* sp. on the tonsillar surface (Figure 4).

**Table 1.** Characteristics of patients with immunoglobulin a nephropathy positive and negative for *Neisseria* sp. on the tonsillar surface who underwent tonsillectomy (*n* = 81).

	*Neisseria* sp (+) (*n* = 71)	*Neisseria* sp (−) (*n* = 10)	*p*-Value
Age	39 ± 15 (35–43)	49 ± 17 (37–61)	0.05
Male	42 (59%)	6 (60%)	>0.99
Duration from diagnosis to tonsillectomy (years)	5 ± 7 (3–7)	8 ± 9 (1–14)	0.26
History of tonsillitis	3 (4%)	1 (10%)	0.42
Coexistence of HT	33 (46%)	3 (30%)	0.50
Usage of RAS inhibitors	26 (37%)	3 (30%)	>0.99
Antibiotic use within 1 month before surgery	0 (0%)	0 (0%)	NA
Serum creatinine levels (mg/dL)	0.87 ± 0.32 (0.79–0.94)	0.91 ± 0.38 (0.63–1.18)	0.72
eGFR (mL/min/1.73 m^2^)	80 ± 27 (73–86)	75 ± 30 (54–96)	0.59
Serum IgA levels (mg/dL)	331 ± 106 (306–356)	322 ± 155 (212–433)	0.81
Qualitative findings of hematuria	1.9 ± 1.0 (1.7–2.2)	2.0 ± 1.0 (1.2–2.7)	0.92
Qualitative findings of proteinuria	1.3 ± 0.8 (1.1–1.5)	1.5 ± 1.0 (0.7–2.2)	0.62
Daily urinary protein extraction (g/day)	0.8 ± 0.9 (0.5–1.0)	1.2 ± 1.1 (0.5–2.5)	0.19
<0.3	26 (37%)	2 (20%)	0.10
0.3–0.4	10 (14%)	2 (20%)	
0.5–1.0	16 (23%)	0 (0%)	
1.0–1.9	12 (17%)	3 (30%)	
>2.0	7 (10%)	3 (30%)	
Oxford classification*			
M1	5 (36%)	0 (0%)	>0.99
E1	2 (14%)	0 (0%)	>0.99
S1	12 (86%)	1 (50%)	>0.99
T1	3 (21%)	0 (0%)	>0.99
T2	0 (0%)	0 (0%)	
H-grade*			
Grade I	36 (68%)	3 (43%)	0.14
Grade II	13 (25%)	2 (29%)	
Grade III	4 (8%)	2 (29%)	
Grade IV	0 (0%)	0 (0%)	
Administration of steroid pulse therapy	50 (70%)	6 (60%)	0.49
Follow-up period (months)	86 ± 74 (68–103)	111 ± 80 (53–168)	0.32

C-grade: clinical-grade; eGFR: estimated glomerular filtration rate; H-grade: histological grade based on the Japanese clinical guidelines for immunoglobulin A nephropathy; HT: hypertension; IgA: immunoglobulin A; NA: not applicable; RAS: renin-angiotensin system.

Data are presented as the mean ± standard deviation (95% confidence interval), or number (percentage).

*Oxford classification data were available for 23 patients, and H-grade data were available for 60 patients.

**Table 2.** Cox proportional-hazards model for factors affecting clinical remission in patients with immunoglobulin a nephropathy who underwent tonsillectomy (*n* = 81).

	Hazard ratio	95% CI	*p*-Value
Age ≥ 40 years	0.91	0.49–1.66	0.75
Usage of RAS inhibitors	0.59	0.30–1.11	0.11
eGFR ≥ 60	0.59	0.30–1.26	0.16
Daily urinary protein extraction ≥ 0.5 (g/day)	0.68	0.37–1.22	0.19
Administration of steroid pulse therapy	2.07	1.10–4.12	0.03*
Positive for *Neisseria* sp. on the tonsillar surface	3.42	1.23–14.25	0.04*

CI: confidence interval; eGFR: estimated glomerular filtration rate; RAS: renin-angiotensin system.

*Statistically significant.

**Table 3.** Cox proportional-hazards model for factors affecting decreased eGFR (30%) in patients with immunoglobulin a nephropathy who underwent tonsillectomy (*n* = 81).

	Hazard ratio	95% CI	*p*-Value
Daily urinary protein extraction ≥ 0.5 (g/day)	23.0	1.76–301	0.05
Administration of steroid pulse therapy	6.42	0.99–41.6	<0.01*
Positive for *Neisseria* sp. on the tonsillar surface	0.03	0.01–0.21	<0.01*

CI: confidence interval; eGFR: estimated glomerular filtration rate.

*Statistically significant.

**Table 4.** Cox proportional-hazards model for factors affecting renal death in patients with immunoglobulin a nephropathy who underwent tonsillectomy (*n* = 81).

	Hazard ratio	95% CI	*p*-Value
Usage of RAS inhibitors	8.14	0.57–115	0.12
Daily urinary protein extraction ≥ 0.5 (g/day)	1.06 × 10^9^	0.00–Inf	0.99
Positive for *Neisseria* sp. on the tonsillar surface	0.06	0.01–0.79	0.03*

CI: confidence interval: inf: infinity: RAS: renin-angiotensin system.

*Statistically significant.

Differences in the treatment outcomes of tonsillectomy between patients with IgAN in the presence or absence of *Neisseria* sp. at the tonsillar center are illustrated in Supplemental Figures S2 and S3. Forty-six patients (56.8%) with IgAN were positive for *Neisseria* sp. at the tonsillar center, whereas 35 patients (43.2%) were not. The 10-year renal survival rates in the presence and absence of *Neisseria* sp. were 100 and 92.9%, respectively (Supplemental Figure S3(b)). The log-rank test revealed that the renal survival rate was significantly higher in the positive group than in the negative group. However, there were no other significant differences in treatment outcomes following tonsillectomy between patients exhibiting the presence and absence of *Neisseria* sp. at the tonsillar center (Supplemental Figures S2 and S3(a)).

## Discussion

The involvement of specific bacteria in the pathogenesis of IgAN was first reported by Suzuki et al. [15]. They demonstrated a significantly higher detection rate of *H. parainfluenzae* in IgAN than in other glomerular diseases using the swab culture method, and reported that tonsillar lymphocytes in IgAN produced higher levels of anti-*H. parainfluenzae*-specific IgA [15]. However, we did not observe a difference in the detection rate of *H. parainfluenzae* between the IgAN and RT groups. Regarding other specific bacteria, results vary among studies [15–23]. Two studies demonstrated that the detection rate of *Treponema* sp. was significantly higher in the tonsillar tissue of patients with IgAN using 16S ribosomal RNA (rRNA) sequencing and denaturing gradient gel electrophoresis (DGGE) [16,17]. Two studies using DGGE and polymerase chain reaction as detection methods reported that *Campylobacter rectus* was predominantly detected in the saliva of patients with IgAN [16,20]. In contrast, our study demonstrated that the detection rate of *Neisseria* sp. on the tonsillar surface was significantly higher in the IgAN group than in the RT group. Currie et al. [18] reported that, using 16S rRNA sequencing, the most frequently detected genus on the tonsillar surface of patients with IgAN was *Neisseria* and its detection rate was significantly higher in patients with IgAN than in controls. This study also demonstrated that *Neisseria*-specific serum IgA levels were elevated in patients with IgAN. In an IgAN mouse model, mucosal infection with *Neisseria* sp. augmented the levels of serum *Neisseria*-specific IgA and the deposition of IgA in kidney tissues. Another study on the bacterial flora in saliva using 16S rRNA sequence also described the predominance of *Neisseria* sp. in patients with IgAN [22]. Several studies have investigated whether the presence of specific bacteria influences the clinical course of IgAN. The presence of *C. rectus* in saliva samples from patients with IgAN was reportedly associated with severe proteinuria and segmental glomerulosclerosis [20], whereas the presence of *Tannerella* sp. was linked to worse kidney function [19]. However, studies including patients with IgAN who underwent tonsillectomy have yielded contradictory results. The presence of *Treponema* sp. or *C. rectus* in tonsillar tissue exhibited favorable clinical remission rates [16]. Additionally, our study is the first to demonstrate that the presence of *Neisseria* sp. on the tonsillar surface is associated with a favorable clinical course, including short- and long-term outcomes, in patients with IgAN after tonsillectomy.

Differences in the bacterial flora between the tonsillar surface and center have been reported in RT [24–27]. The detection rates of bacteria at the tonsillar center are reportedly higher than those on the surface [24,25]. Other studies have revealed that pathogenic organisms are mainly detected at the tonsillar center, whereas commensal bacteria are detected on the surface [26,27]. These studies indicate that the presence of pathogenic bacteria at the tonsillar center may cause the recurrence of tonsillitis in patients with RT. Conversely, our study revealed that the presence of bacteria at the tonsillar center might not be associated with the clinical course of IgAN. The detection rate of *Neisseria* sp. at the tonsillar center in patients with IgAN was comparable to that in patients with RT. Additionally, the presence of *Neisseria* sp. at the center could predict renal survival rates, but not clinical remission, negative conversion of hematuria and proteinuria, or preservation of renal function in patients with IgAN. These results indicate that the bacteria on the tonsillar surface are more accurately related to the clinical course of IgAN. The difference between the tonsillar surface and center in IgAN might be attributed to the anatomical structure of the tonsils. The formation of deep tubular crypts on the tonsillar surface contributes to the expansion of the tonsillar surface area, resulting in more effective contact with foreign antigens. Antigen-presenting cells such as membranous epithelial cells and dendritic cells are distributed in the lymphoid epithelial symbiosis of the tonsillar crypts [28]. Thus, the tonsillar surface, particularly the tonsillar crypts, is believed to be an important site for tonsillar immune cells to react with microorganisms in IgAN.

In this study, no significant difference was observed in the serum IgA levels based on the presence or absence of *Neisseria* sp. on the tonsillar surface. It is known that serum IgA levels are not always elevated in IgAN [29], and glomerular damage does not necessarily occur even if IgA is deposited [30,31]. These findings suggest that IgAN is not solely driven by excessive IgA quantity, which may explain the lack of a significant difference in serum IgA levels between the two groups. Previous studies have revealed that patients with IgAN exhibit elevated levels of galactose-deficient IgA1 (Gd-IgA1), a qualitatively abnormal form of IgA that plays a key role in renal injury [29]. Gd-IgA1 has also been implicated in the formation of pathogenic immune complexes in renal tissue. One hypothesis suggests that abnormal mucosal immune responses to exogenous antigens lead to the production of qualitatively altered IgA, such as Gd-IgA1, which subsequently forms immune complexes *via* interaction with autoantibodies. On the other hand, another hypothesis proposes that, in response to exogenous antigens, antigen-specific IgA is produced and directly forms immune complexes with these antigens [32,33]. Previous studies have reported the co-deposition of IgA with bacterial antigens such as *S. aureus* [34] and *H. parainfluenzae* [15] in renal tissue. In this study, we investigated the presence of *Neisseria* antigens in renal tissue using immunohistochemical staining, and observed positive staining of *N. meningitidis* in the glomeruli. Although these findings may support the latter hypothesis, positive staining was similarly observed in patients without *Neisseria* sp. on the tonsillar surface. Due to the limited number of available renal biopsy specimens, further studies are needed to clarify the presence and significance of bacterial antigens in renal tissue.

This study had several limitations. First, specific bacteria, in particular anaerobes, were underestimated due to the swab culture detection methods. The differences in bacterial flora between our study and other studies may be attributable to detection methods or specimen collection sites. Previous studies have obtained bacterial specimens from various sites including tonsillar surface [17,18], whole tonsil tissues [16], surface of the pharynx [15], and saliva [20,22]. Additionally, a shortcoming of the swab culture method based on conventional phenotypic methods is that the identification of the specific *Neisseria* sp. was not feasible. The *Neisseria* genus includes several species such as *Neisseria gonorrhoeae*, *meningitidis*, *subflava*, *mucosa*, and *sicca*. In this study, it remains unclear which *Neisseria* sp. are associated with the clinical course of IgAN. Furthermore, inclusion of all *Neisseria* sp. in the immunohistochemical analysis was not possible due to the lack of specific antibodies. 16S rRNA sequencing identifies specific bacteria by analyzing the 16S rRNA gene, which is universally present among bacteria. This method offers high sensitivity and specificity, allowing for more accurate and detailed detection of bacterial species. We are currently conducting 16S rRNA sequencing of tonsillar tissue to provide a more comprehensive microbiome analysis. However, 16S rRNA sequencing has not yet been applied in routine clinical practice, and in reality, diagnostic testing still largely relies on culture-based methods. From a clinical perspective, the results of our swab-based analysis may therefore be considered more practical and applicable to real-world settings. Second, only 10 patients in our study were *Neisseria*-negative on the tonsillar surface, which may limit the strength of our conclusions. Further studies with larger sample sizes are warranted to clarify these findings. Third, decreased renal function and renal death occurred in only 11 and 4 patients, respectively. Thus, we applied a stepwise method in the Cox proportional hazards models to optimize the explanatory variables, and the models revealed that the presence of *Neisseria* sp. on the tonsillar surface was an independent favorable predictor of these events. However, the models could potentially lack sufficient accuracy and consistency. Fourth, although both the present and previous studies suggest that an excessive immune response to *Neisseria* sp. may play a role in the pathogenesis of IgAN, our study did not provide direct evidence that *Neisseria* sp. are involved in the disease mechanism. We are planning a stimulation experiment of *Neisseria* sp. on tonsillar mononuclear cells from patients with IgAN to provide direct evidence.

In conclusion, this study revealed that the detection rate of *Neisseria* sp. on the tonsillar surface was significantly higher in patients with IgAN than in those with RT. Moreover, this study demonstrated that patients with IgAN with the presence of *Neisseria* sp. on the tonsillar surface experienced a favorable clinical course after tonsillectomy.

## Supplementary Material

Supplemental Material

Supplemental Material

Supplemental Material
