# Supplementary figures and images for: Neisseria species on the tonsillar surface predicts favorable clinical outcomes in patients with Immunoglobulin A nephropathy who underwent tonsillectomy
Source: Ren Fail. 2025 Sep 3;47(1):2550617. doi: 10.1080/0886022X.2025.2550617 (PMC12409855; doi:10.1080/0886022X.2025.2550617)

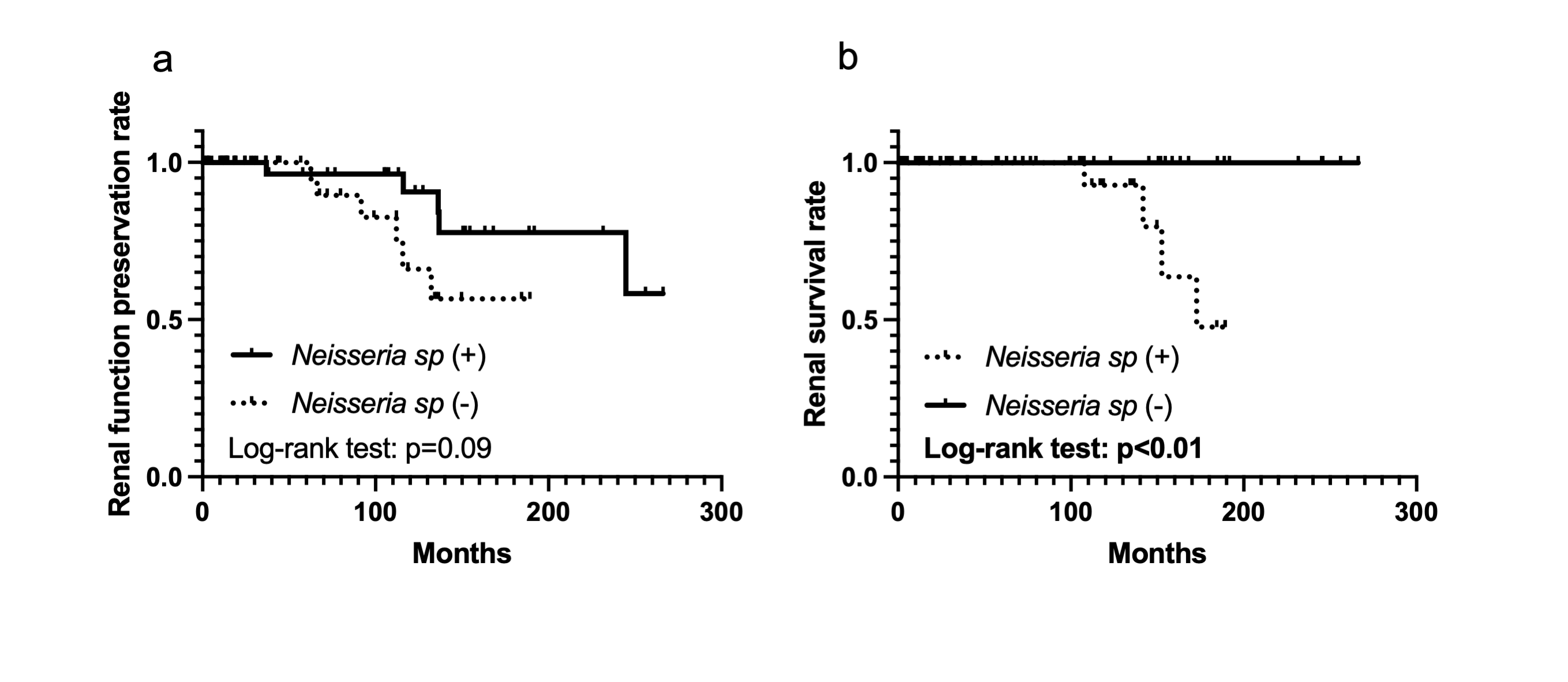

Supplement: Supplemental Material [file IRNF_A_2550617_SM3153.tiff]

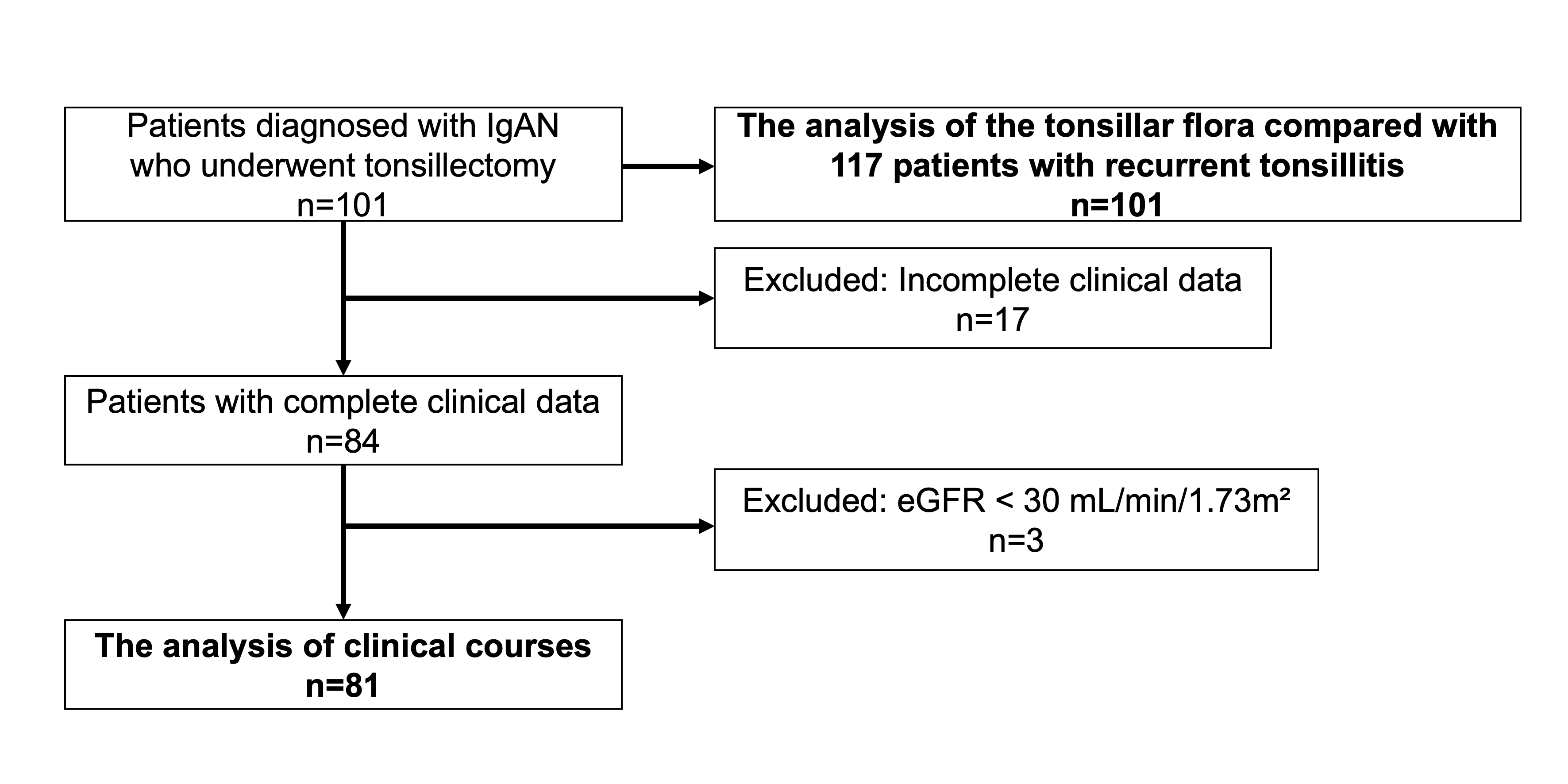

Supplement: Supplemental Material [file IRNF_A_2550617_SM3151.tiff]

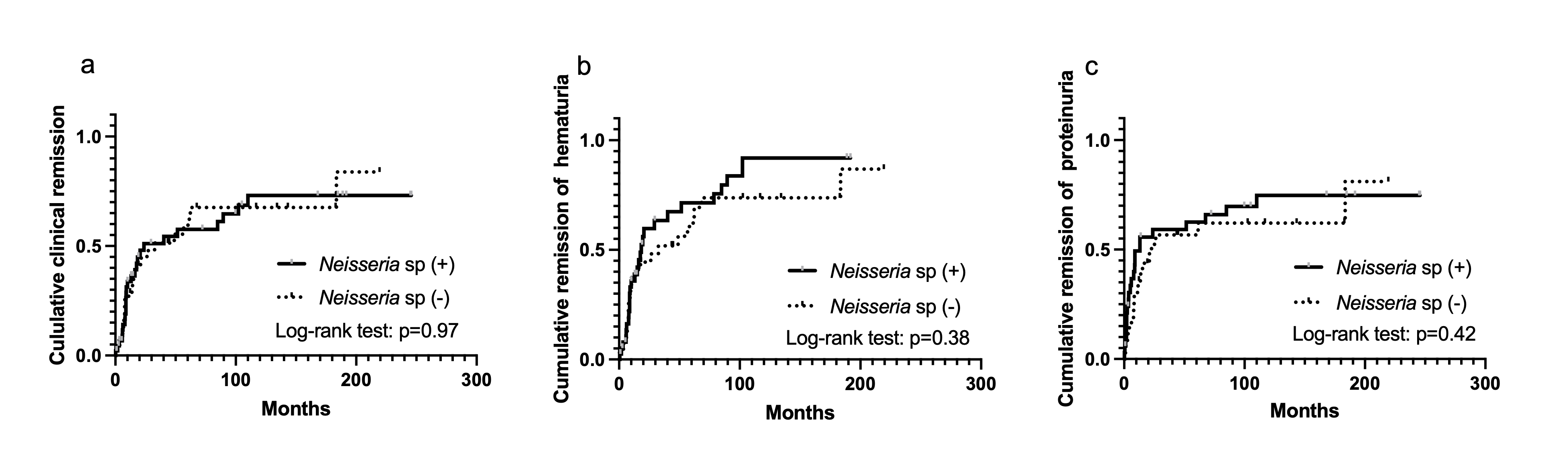

Supplement: Supplemental Material [file IRNF_A_2550617_SM3148.tiff]
